# YAP Inhibition by Resveratrol via Activation of AMPK Enhances the Sensitivity of Pancreatic Cancer Cells to Gemcitabine

**DOI:** 10.3390/nu8100546

**Published:** 2016-09-23

**Authors:** Zhengdong Jiang, Xin Chen, Ke Chen, Liankang Sun, Luping Gao, Cancan Zhou, Meng Lei, Wanxing Duan, Zheng Wang, Qingyong Ma, Jiguang Ma

**Affiliations:** 1 Department of Hepatobiliary Surgery, First Affiliated Hospital of Xi’an Jiaotong University, Xi’an 710061, China; jiang.19900708@stu.xjtu.edu.cn (Z.J.); chenxin.77@stu.xjtu.edu.cn (X.C.); ck532128@stu.xjtu.edu.cn (K.C.); sunliankang@stu.xjtu.edu.cn (L.S.); gaoluping@stu.xjtu.edu.cn (L.G.); trytofly@stu.xjtu.edu.cn (C.Z.); leimeng7@stu.xjtu.edu.cn (M.L.); 0556029@fudan.edu.cn (W.D.); zheng.wang11@mail.xjtu.edu.cn (Z.W.); 2 Department of Anesthesiology, First Affiliated Hospital of Xi’an Jiaotong University, Xi’an 710061, China

**Keywords:** pancreatic cancer, resveratrol, AMPK, YAP, gemcitabine

## Abstract

Resveratrol, a natural polyphenol present in most plants, inhibits the growth of numerous cancers both in vitro and in vivo. Aberrant expression of YAP has been reported to activate multiple growth-regulatory pathways and confer anti-apoptotic abilities to many cancer cells. However, the role of resveratrol in YES-activated protein (YAP) expression and that of YAP in pancreatic cancer cells’ response to gemcitabine resistance remain elusive. In this study, we found that resveratrol suppressed the proliferation and cloning ability and induced the apoptosis of pancreatic cancer cells. These multiple biological effects might result from the activation of AMP-activation protein kinase (AMPK) (Thr172) and, thus, the induction of YAP cytoplasmic retention, Ser127 phosphorylation, and the inhibition of YAP transcriptional activity by resveratrol. YAP silencing by siRNA or resveratrol enhanced the sensitivity of gemcitabine in pancreatic cancer cells. Taken together, these findings demonstrate that resveratrol could increase the sensitivity of pancreatic cancer cells to gemcitabine by inhibiting YAP expression. More importantly, our work reveals that resveratrol is a potential anticancer agent for the treatment of pancreatic cancer, and YAP may serve as a promising target for sensitizing pancreatic cancer cells to chemotherapy.

## 1. Introduction

Pancreatic ductal adenocarcinoma (PDAC) is one of the most malignant and lethal tumors, with an overall five-year survival rate less than 7% [1]. Over recent decades, the prognosis of patients with this malignancy has not improved due to aggressive local invasion, metastases, and resistance to chemotherapy [2]. Currently, surgical resection is the only opportunity for curing pancreatic cancer at an early stage. Unfortunately, only approximately 20% of patients are eligible for surgical resection at the time of diagnosis, with most patents losing the opportunity for radical surgery. At present, gemcitabine is the first-line chemotherapeutic agent for pancreatic cancer patients. However, a low response rate to gemcitabine is common in the clinic, and less than 20% of patients experience the ideal effects of gemcitabine [3]. In recent years, FOLFIRINOX (oxaliplatin, irinotecan, fluorouracil, and leucovorin) has become the recommended frontline chemotherapeutic regimen for metastatic pancreatic cancer patients [4]. However, the seriously adverse reaction and acquired drug resistance associated with FOLFIRINOX limits its cytotoxic efficacy. Therefore, a novel target for enhancing current chemotherapy is clearly needed to improve the outcomes of patients with pancreatic cancer.

The Hippo pathway was first discovered by genetic mosaic screens in *Drosophila melanogaster* [5,6], and increasing evidence has demonstrated that the Hippo pathway also limits organ size in mammalian systems [7,8]. The YES-associated protein (YAP), a main component of the Hippo pathway, has been proved to be overexpressed and to participate in the tumorigenesis of a variety of cancers, including breast cancer [9], lung cancer [10], ovarian cancer [11], liver cancer [12], and pancreatic cancer [13,14]. YAP functions as an oncogene and promotes the survival of cancer cells by regulating cancer cell proliferation and apoptosis. The abnormal overexpression of YAP has also been reported to be linked to disease progression and poor prognosis in breast cancer patients [15]. However, whether aberrant YAP expression causes resistance of pancreatic cancer cells to chemotherapy is currently unclear. We hypothesized that overexpression of YAP might be closely correlated to the sensitivity of gemcitabine treatment in pancreatic cancer cells.

Resveratrol (*trans-3,4',5-trihydroxystilbene*, Res) is a natural polyphenolic phytoalexin that was first isolated by Takaoka from the roots of white hellebore in 1939. Since then, resveratrol has been widely found in plants (such as grape skin, red wine, berries, and peanuts) and in traditional Chinese medicines (such as Rheum officinale Baill and Polygonum cuspidatum) [16,17]. Numerous studies have shown that resveratrol exhibits antioxidant activity, anti-inflammatory activity, and protective activity against cardiac diseases and metabolic disorders [18]. Over the past several years, many reports have demonstrated that resveratrol acts as a cancer chemo-preventive agent to induce growth inhibition, cell cycle arrest, apoptosis, and changes in biomarker expression in several human cancer cell lines [19,20,21]. Our previous study showed that resveratrol plays an important role in suppressing the proliferation of pancreatic cancer cells via the PI-3K/Akt/NF-κB signaling pathway [22] and the hedgehog signaling pathway [23,24]. However, whether resveratrol can inhibit YAP expression and its molecular mechanism have not been elucidated.

In the present study, we tested the hypothesis that resveratrol is able to inhibit YAP expression via activation of the AMP-activated protein kinase (AMPK) pathway and inhibit the proliferation ability of pancreatic cancer cells. YAP is associated with resistance to gemcitabine chemotherapy and targeting YAP via resveratrol can enhance the sensitivity of pancreatic cancer cells to gemcitabine. Our findings indicate that YAP is a novel molecular target for improving the efficacy of current chemotherapeutic regimens for patients with pancreatic cancer and improving their long-term outcomes.

## 2. Materials and Methods

All experimental protocols were approved by the Ethical Committee of the First Affiliated Hospital of Medical College, Xi’an Jiaotong University, Xi’an, China.

### 2.1. Reagents

Resveratrol (>99% pure) and MTT (3-(4,5-dimethyl-2-thiazolyl)-2,5-diphenyl-2-H-tetrazolium bromide) were purchased from Sigma-Aldrich (St. Louis, MO, USA), and gemcitabine was purchased from Selleck Chemicals (Houston, TX, USA). Resveratrol and gemcitabine were initially dissolved in Dimethyl Sulfoxide (DMSO) at stock concentrations of 50 mM and 10 mM, respectively. Working dilutions for resveratrol and gemcitabine were prepared in culture medium immediately before use, and DMSO was used as control in all experiments. The antibodies used in this study are listed in Supplementary Materials Table S1.

### 2.2. Cell Lines and Cell Culture

The human pancreatic cancer cell lines Panc-1 and BxPC-3 were purchased from the Type Culture Collection of the Chinese Academy of Sciences (Shanghai, China). Panc-1 and BxPC-3 were cultured in Dulbecco's Modified Eagle Medium (DMEM) or RPMI-1640, respectively, containing 10% dialyzed heat-inactivated fetal bovine serum (FBS) (HyClone, Logan, UT, USA), 100 U/mL penicillin and 100 μg/mL streptomycin in a humidified atmosphere containing 5% CO_2_ at 37 °C.

### 2.3. Cell Viability Assay

Cancer cell lines (Panc-1, BxPC-3) were plated into 96-well plates at a density of 5000 cells/well and treated with various concentrations (0, 25, 50, 100, and 200 μM) of resveratrol and various concentrations (0, 1, 2, 5, 10, and 20 μM) of gemcitabine for designated lengths of time (24, 48, and 72 h). After being transfected with siRNA for 48 h, cells were plated in 96-well plates at a density of 5000 cells/well and treated with 2 μM gemcitabine for 72 h. Cell viability was assessed by the MTT assay. Ten microliters of 5 mg/mL MTT was added into each well after media were removed and incubated at 37 °C for 4 h. Then, 100 μL DMSO was added to each well, and the optical density (OD) was measured at 490 nm on a multifunction microplate reader (POLARstar OPTIMA; BMG, Offenburg, Germany). The proliferation inhibition rate was calculated according to the following equation: Proliferation inhibition rate = (1 − OD sample/OD control) × 100%.

### 2.4. Apoptosis Assay

Cell apoptosis was assessed by flow cytometry with an Annexin V-FITC/7-AAD apoptosis detection kit from Becton, Dickinson and Company (BD) (Franklin Lakes, NJ, USA) according to the manufacturer’s instructions. Briefly, cancer cells were seeded into 6-well plates at a density of 1 × 10^5^ cells per well, after being starved overnight, and each treatment was applied for 48 h. Then, cells were trypsinized, washed with phosphate buffered saline (PBS) and stained with Annexin V and 7-AAD. The percentage of apoptotic cells was quantified by flow cytometry using a FACSCalibur (BD Biosciences, San Diego, CA, USA) instrument. The total apoptosis rate was calculated by summing the rate of populations stained with Annexin V-FITC+/7-AAD- (early apoptotic cells) and Annexin V-FITC+/7-AAD+ (late apoptotic cells).

### 2.5. Immunofluorescence Staining

Cells were fixed in 4% formaldehyde diluted in phosphate buffered saline (PBS) for 15 min, permeabilized with 0.3% Triton X-100, treated with blocking buffer (5% BSA in PBS), and then incubated overnight with the primary antibody at 4 °C. Cells were then incubated with the Red conjugated secondary antibody from Jackson Immunoresearch Laboratories (West Grove, PA, USA) for 1 h at room temperature. Slides were mounted and examined using a Zeiss Instruments confocal microscope.

### 2.6. Gene Silencing by Small Interfering RNA

Loss-of-function analysis was performed using siRNAs targeting AMPK and YAP, which were purchased from GenePharm (Shanghai, China). The siRNA sequences are provided in Supplementary Materials Table S2. Each siRNA (100 nM) was transfected into pancreatic cancer cells using Lipofectamine 2000 according to the manufacturer’s instructions. The knockdown of each target gene was confirmed by Western blot analysis. The cells were used for subsequent experiments 48 h after transfection.

### 2.7. Western Blot Analysis

Total proteins were extracted by RIPA lysis buffer (Beyotime, Guangzhou, China), and the concentration of proteins was determined using the BCA protein assay kit (Pierce, Rockford, IL, USA) according to the manufacturer’s instruction. The proteins were then subjected to SDS-PAGE using a 10% polyacrylamide gel with a 5% stacking gel. The proteins were subsequently transferred to polyvinylidene difluoride (PVDF) membranes. The membranes were blocked with 5% fat-free milk in Tris-buffered saline-Tween (TBS-T) for 2 h and then incubated with the primary antibodies (listed in Supplementary Materials Table S1) at 4 °C overnight. Then, the membranes were incubated with a secondary antibody (diluted 1:10,000) for 2 h at room temperature. Chemiluminescence detection of bound antibodies was performed using an enhanced chemiluminescence (ECL) PLUS system and a Molecular Imager ChemiDoc XRS System (Bio-Rad Laboratories, Hercules, CA, USA).

### 2.8. Real-Time PCR

Total RNA was extracted using the Fastgen1000 RNA isolation system (Fastgen, Shanghai, China) according to the manufacturer’s protocol. Total RNA was reverse-transcribed into cDNA using the Prime Script RT reagent kit (TaKaRa, Dalian, China). Real-time PCR was used to quantitatively examine the expression of YAP, CTGF and CYR61 at the mRNA level. Real-time PCR was conducted according to a previous report [25]. The PCR primer sequences for YAP, CTGF, CYR61, and β-actin are shown in Supplementary Materials Table S3. The expression of each target gene was determined using β-actin as the normalization control. Relative gene expression was calculated using the 2^−^^ΔΔCt^ method [26].

### 2.9. Colony Formation Assay

One thousand cells were seeded into a 35-mm petri dish and allowed to adhere overnight. The next day, a different treatment was applied to the dishes for 24 h, after which the medium was replaced with drug-free medium. Cells were further cultured for two weeks to allow colonies to form. At the indicated time point, colonies were fixed with 4% paraformaldehyde and then stained with 0.1% crystal violet solution, rinsed, and then imaged. The number of colonies larger than 0.5 mm in diameter was counted using a microscope (Nikon Eclipse Ti-S, Tokyo, Japan) at a magnification of 400×.

### 2.10. Statistical Analysis

Each experiment was performed at least three times. Data are presented as means ± standard deviation. Differences were evaluated using Student’s *t*-test, with *p* < 0.05 considered to be statistically significant.

## 3. Results

### 3.1. Resveratrol Inhibits the Proliferation of Pancreatic Cancer Cells

**Figure 1:**
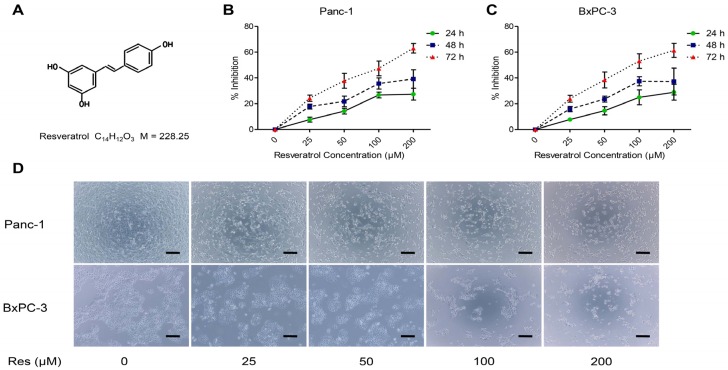
Resveratrol inhibits the proliferation of pancreatic cancer cells. (**A**) The structure of resveratrol (Res); (**B**,**C**) Panc-1 and BxPC-3 pancreatic cancer cells were treated with increasing concentrations of resveratrol (0, 25, 50, 100, and 200 μM) for 24 h, 48 h, or 72 h to analyze the inhibition ratio for cancer cell proliferation; (**D**) Micrographs of Panc-1 and BxPC-3 cells after being treated with the indicated concentrations of resveratrol for 48 h; representative images were captured (magnification, 100×; Scar bar: 100 μm).

First, we examined the effects of resveratrol on the viability of cancer cells. Pancreatic cancer cells Panc-1 and BxPC-3 were treated with increasing doses of resveratrol (0, 25, 50, 100, and 200 μM). At the indicated time points (24, 48, and 72 h), the cell viability was assessed by the MTT assay. As shown in Figure 1, resveratrol decreased the growth of cancer cell lines in a dose- and time-dependent manner. The 50% inhibitory concentration (IC50) for both BxPC-3 and Panc-1 cells was approximately 50 μM resveratrol, which exhibited no cytotoxic effects on the BxPC-3 and Panc-1 cells. These results were in accord with our previous results. Therefore, cells were treated with 50 μM resveratrol in subsequent experiments.

### 3.2. Resveratrol Inhibits Clone Formation and Induces Apoptosis of Pancreatic Cancer Cells

**Figure 2:**
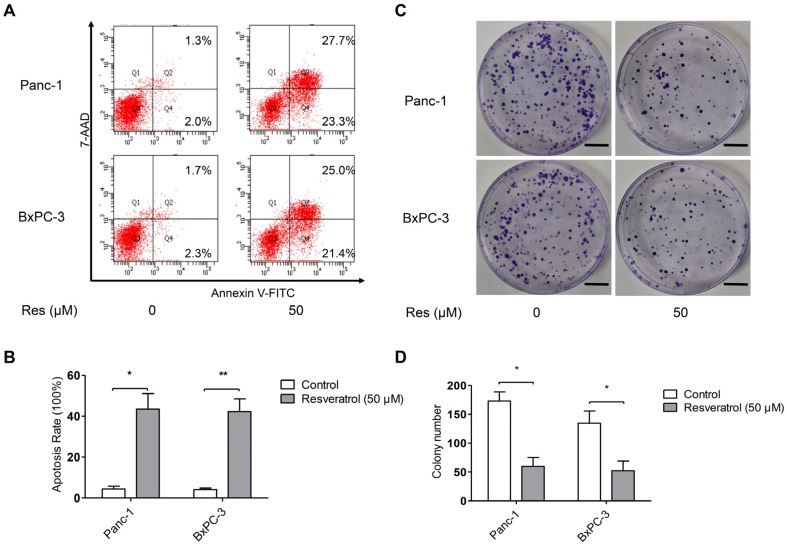
Resveratrol inhibits clone formation and induces apoptosis of pancreatic cancer cells. (**A**,**B**) The effects of resveratrol on Panc-1 and BxPC-3 cells apoptosis were detected by flow cytometry; (**C**,**D**) The effects of resveratrol on the colony-forming ability of Panc-1 and BxPC-3 cells. Images are representative of three independent experiments (Scale bar: 1 cm). Column: Mean, bar: SD, * *p* < 0.05, ** *p* < 0.01.

To address the underlying mechanism governing the inhibitory effect of resveratrol (Res) on pancreatic cancer cell viability, we measured Res-induced apoptosis in BxPc-3 and Panc-1 cells by flow cytometry. The flow cytometric analyses were conducted after Panc-1 and BxPC-3 cells were treated with or without resveratrol (50 μM) for 48 h. As shown in Figure 2A,B, treatment of cancer cells with resveratrol caused an increase in the apoptotic population compared with that of the untreated control cells. Next, we detected the effect of resveratrol on the clone formation ability of cancer cells Panc-1 and BxPC-3. As shown in Figure 2C,D, treatment with 50 μM resveratrol markedly decreased the number of colonies compared with the untreated control cells. These results demonstrate that Res has a potent effect against clone formation and induces apoptosis of cancer cells.

### 3.3. Resveratrol Inhibits YAP Expression of Pancreatic Cancer Cells

**Figure 3:**
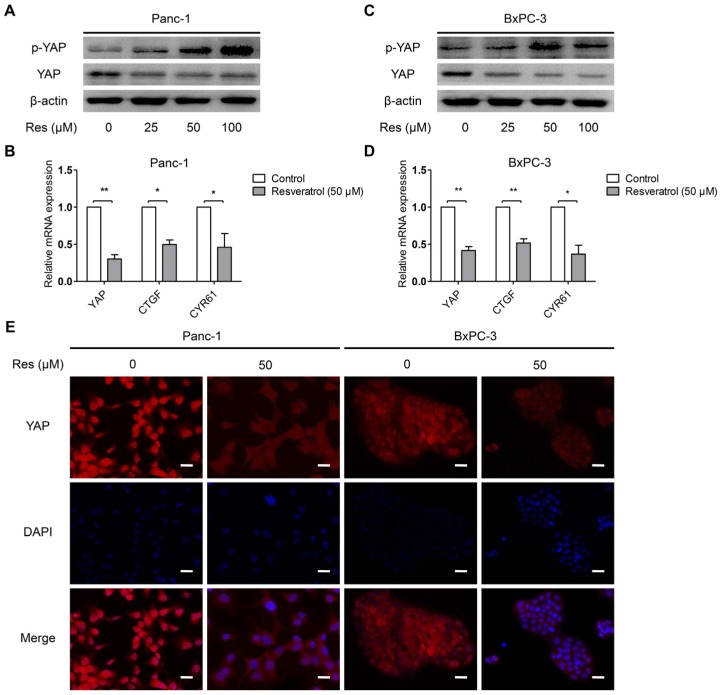
Resveratrol inhibits YAP expression of pancreatic cancer cells. (**A**,**B**) The effects of resveratrol on the protein expression of YAP and p-YAP (Ser127) were examined by Western blot analysis using β-actin as an internal loading control; (**C**,**D**) The effects of resveratrol on the mRNA expression of YAP, connective tissue growth factor (CTGF) and CYR61 were examined by real-time PCR with β-actin as the normalized reference gene. The data represent the results of three independent experiments; (**E**) Immunofluorescence staining of YAP in Panc-1 and BxPC-3 cells after treatment with resveratrol (50 μM) for 24 h. YAP staining is shown in red, and nuclear DNA staining by DAPI is shown in blue. (Magnification, 400×; Scale bar: 20 μm). Column: mean, bar: SD, * *p* < 0.05, ** *p* < 0.01.

Increasing evidence has suggested that overexpression of YAP plays a key role in cancer cell survival and progression [27]. In particular, YAP can be phosphorylated at Ser127 and forms a more stable complex with the 14-3-3 proteins; therefore, it is retained in the cytoplasm and subject to degradation [28]. To determine whether resveratrol affects the YAP expression of cancer cells, Panc-1 and BxPC-3 cells were treated with resveratrol (0, 25, 50, and 100 μM) for 24 h. The protein expression of YAP and p-YAP (Ser127) in the pancreatic cancer cells exposed to resveratrol was evaluated by Western blot analysis. As shown in Figure 3A,B, resveratrol treatment up-regulated the level of p-YAP (Ser127), and the total level of YAP was significantly inhibited by resveratrol in a dose-dependent manner. Connective tissue growth factor (CTGF) and cysteine-rich angiogenic inducer 61 (CYR61) are two YAP-mediated downstream effectors that play an important role in tumor progression [29,30]. We therefore examined the expression of YAP, CTGF and CYR61 in response to treatment with resveratrol. The results showed that the mRNA levels of YAP, CTGF, and CYR61 were downregulated upon treatment with resveratrol (50 μM) (Figure 3C,D). Additionally, the nuclear translocation of YAP was decreased due to the effect of resveratrol, as demonstrated by immunofluorescence (Figure 3E). Together, these data indicate that resveratrol inhibits YAP expression of cancer cells via YAP phosphorylation at Ser127.

### 3.4. Knockdown of AMPK Rescues Resveratrol Induced Suppression of YAP in Pancreatic Cancer Cells

**Figure 4:**
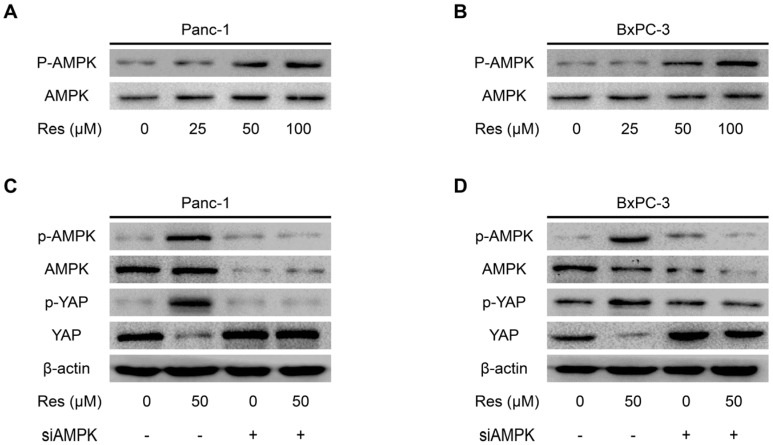

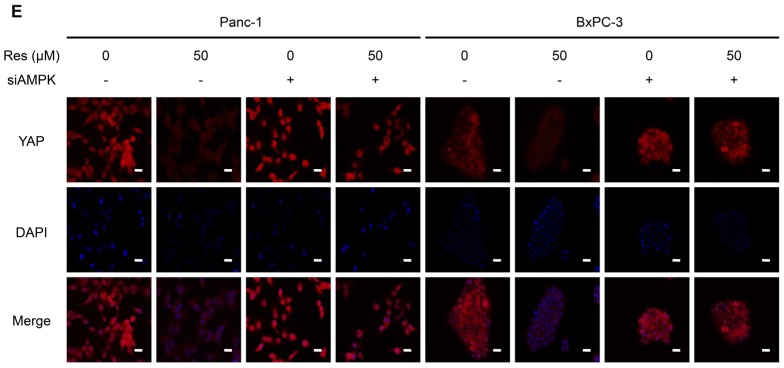
Knockdown of AMP-activated protein kinase (AMPK) rescues resveratrol-induced suppression of YES-associated protein (YAP) of pancreatic cancer cells. (**A**,**B**) The effects of resveratrol on the activation of AMPK (Thr172) in Panc-1 and BxPC-3 cells were measured by Western blot analysis; (**C**,**D**) After transfection with siAMPK or siControl for 48 h, Panc-1 and BxPC-3 cells were treated with resveratrol (50 μM) for 24 h. The protein expression levels of YAP, p-YAP (Ser127), AMPK and p-AMPK (Thr172) were examined by Western blot analysis using β-actin as an internal loading control; (**E**) YAP in Panc-1 and BxPC-3 cells was stained for immunofluorescence after transfection with siAMPK or siControl for 48 h and then treated with resveratrol (50 μM) for 24 h. YAP staining is shown in red, and nuclear DNA staining by DAPI is shown in blue (Magnification, 400×; Scale bar: 20 μm).

Previous studies have established that resveratrol can activate the AMPK pathway [31]. The activation of AMPK leads to the suppression of YAP expression [32]. Based on the abovementioned promising findings, we speculated that the effect of resveratrol on cancer cell YAP inhibition may be mediated by AMPK signaling. To test this hypothesis, we further examined the effect of resveratrol on the activity of AMPK signaling. Immunoblotting results revealed that the phosphorylation level of AMPK (p-AMPK) in pancreatic cancer cells was significantly increased in response to resveratrol treatment in a dose-dependent manner (Figure 4A,B). To verify that resveratrol-inhibited YAP expression in cancer cells is mediated by AMPK signaling, siRNA technology was developed to knock down AMPK expression. We found that knocking down AMPK expression alone did not affect the expression of YAP or the phosphorylation level of p-YAP in Panc-1 and BxPC-3 cells (Figure 4C,D). However, resveratrol induced the activation of p-AMPK and p-YAP, and inhibition of YAP was restored by AMPK knockdown (Figure 4C,D). Additionally, the immunofluorescence results indicated that the nuclear translocation and total level of YAP was inhibited by resveratrol and that this inhibition effect was restored by AMPK knockdown (Figure 4E). Together, these data suggest that AMPK signaling is involved in resveratrol-suppressed YAP expression in pancreatic cancer cells.

### 3.5. Knockdown of YAP Increased Gemcitabine Sensitivity in Pancreatic Cancer Cells

**Figure 5:**
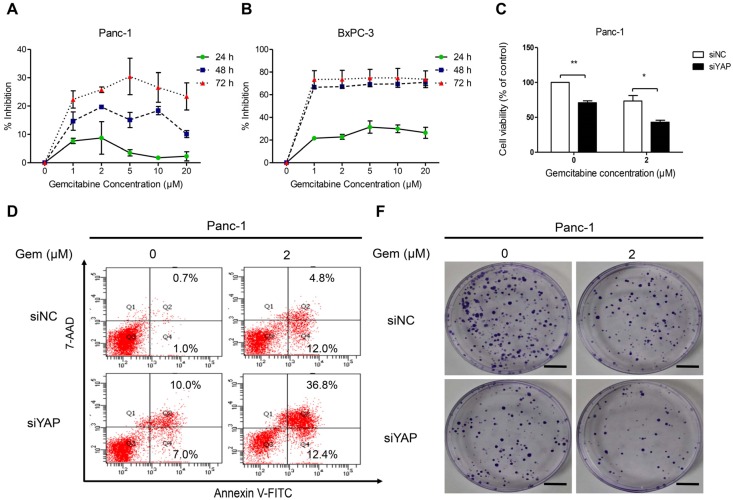

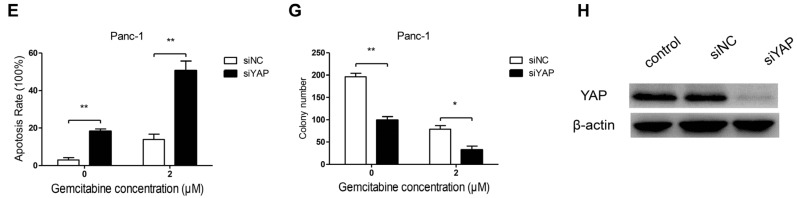
Knockdown of YAP increases gemcitabine sensitivity in pancreatic cancer cells. (**A**,**B**) Panc-1 and BxPC-3 cells were treated with increasing concentrations of gemcitabine (0, 1, 2, 5, 10, and 20 μM) for 24 h, 48 h, or 72 h to analyze the inhibition ratio for cancer cell proliferation; (**C**) After being transfected with siControl or siYAP for 48 h, cells were plated in 96-well plates and treated with 2 μM gemcitabine for 72 h. Cell viability was assessed by the MTT assay; (**D**,**E**) The effects of siControl or siYAP on Panc-1 cancer cells apoptosis after treatment with 2 μM gemcitabine for 48 h was detected by flow cytometry; (**F**,**G**) The effects of siControl or siYAP combined with gemcitabine on the colony-forming ability of Panc-1 cells. Images are representative of three independent experiments (Scale bar: 1 cm); (**H**) The efficiency of siRNAs targeting YAP in Panc-1cells was evaluated by Western blot analysis. Column: Mean, bar: SD, * *p* < 0.05, ** *p* < 0.01.

First, we used the MTT assay to examine the effects of gemcitabine on the proliferation of the Panc-1 and BxPC-3 cell lines. As shown in Figure 5A,B, we found that BxPC-3 was sensitive whereas Panc-1 was resistant to gemcitabine, in accord with previous findings [33]. Therefore, we used Panc-1 in further experiments. To evaluate the effects of YAP on cell survival and resistance to chemotherapy, we treated Panc-1 cells, which express high levels of YAP natively and are resistant to gemcitabine, with 2 μM gemcitabine in the presence of either siControl or siYAP and confirmed the silencing of YAP in the cells using Western blot analysis (Figure 5H). MTT assay results showed that the proliferation capacity was lower in siYAP cells than in siControl cells after treating them with gemcitabine (Figure 5C). Furthermore, silencing of YAP increased the apoptotic response to treatment with gemcitabine (2 μM) in Panc-1 cells (Figure 5D,E). The clone ability was significantly decreased after silencing YAP, and siYAP enhanced the gemcitabine inhibition effect on clone ability (Figure 5F,G). Together, these data suggest that YAP silencing enhances the sensitivity of gemcitabine in gemcitabine-resistant pancreatic cancer cells.

### 3.6. Inhibition of YAP Activity by Resveratrol Enhanced the Sensitivity of Pancreatic Cancer Cells to Gemcitabine

**Figure 6:**
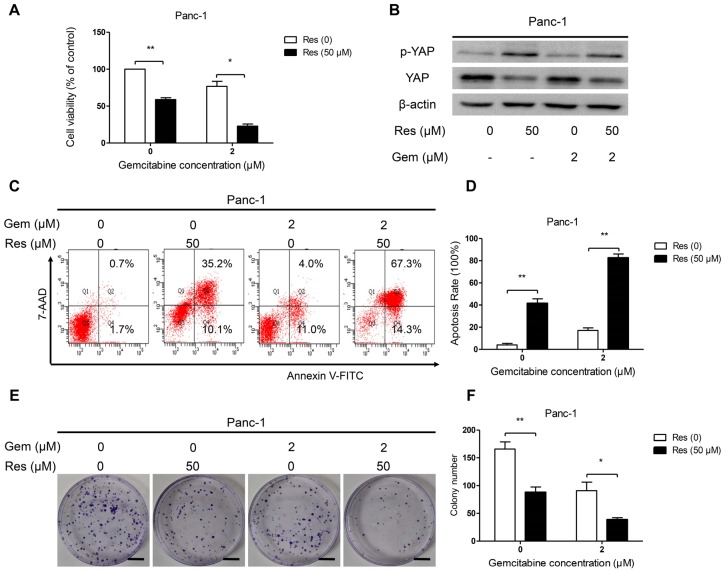
Inhibition of YAP activity by resveratrol enhances the sensitivity of pancreatic cancer cells to gemcitabine. (**A**) Panc-1 cells were treated with gemcitabine (2 μM) and resveratrol (50 μM) for 72 h. Cell viability was assessed by the MTT assay; (**B**) Panc-1 cells were treated with gemcitabine (2 μM) and resveratrol (50 μM) for 24 h. The protein expression levels of YAP and p-YAP (Ser127) were examined by Western blot analysis using β-actin as an internal loading control; (**C**,**D**) Panc-1 cells were treated with gemcitabine (Gem) (2 μM) and resveratrol (50 μM) for 48 h. Apoptosis was detected by flow cytometry; (**E**,**F**) The combined effects of Res and Gem on Panc-1 cell clones’ colony-forming ability was detected. Images are representative of three independent experiments (Scale bar: 1 cm). Column: Mean, bar: SD, * *p* < 0.05, ** *p* < 0.01.

To determine whether resveratrol increases the susceptibility of Panc-1 cells to gemcitabine, we treated Panc-1 cells with 50 μM resveratrol and 2 μM gemcitabine. The MTT assay results showed that the proliferation capacity was significantly lower in the resveratrol and gemcitabine therapy group than in the resveratrol alone or gemcitabine alone group (Figure 6A). The protein expression of YAP and p-YAP (Ser127) in Panc-1 exposed to resveratrol and gemcitabine was evaluated by Western blot analysis. As shown in Figure 6B, resveratrol treatment up-regulated the level of p-YAP (Ser127), and the total level of YAP was significantly inhibited by resveratrol. However, gemcitabine had no effect on YAP expression. Next, we measured the apoptosis rate in Panc-1 cells by flow cytometry. Flow cytometric analyses were conducted after Panc-1 cells were treated with or without resveratrol (50 μM) and gemcitabine (2 μM) for 48 h. As shown in Figure 6C,D, treatment of cancer cells with resveratrol caused an increase in apoptotic population compared with the untreated control cells, but almost all cells underwent apoptosis in the resveratrol and gemcitabine combined therapy group. Next, we detected the clone formation ability of Panc-1 cancer cells after treatment with resveratrol and gemcitabine. As shown in Figure 6E,F, treatment with resveratrol and gemcitabine markedly decreased the number of colonies compared with the number measured for the gemcitabine or resveratrol alone cells. Taken together, these results demonstrate that resveratrol has a potent effect in enhancing the sensitivity of pancreatic cancer cells to gemcitabine by inhibiting YAP expression.

## 4. Discussion

In this study, we observed that resveratrol inhibited pancreatic cancer cell proliferation and clone formation and induced cell apoptosis, which was accompanied by the activation of p-AMPK and p-YAP and a decreased level of YAP in BxPc-3 and Panc-1 cells. Moreover, the results suggest that AMPK signaling is essential in resveratrol-suppressed YAP expression in pancreatic cancer cells. Furthermore, we found that YAP silencing enhances the sensitivity of gemcitabine in gemcitabine-resistant Panc-1 cancer cells. Resveratrol has a synergistic effect with gemcitabine in Panc-1 cells by inhibiting YAP expression. Our findings suggest that resveratrol is a potential drug for adjuvant therapy or a complementary alternative medicine for the management of pancreatic cancers via activation of AMPK and inhibition of YAP.

Despite great advances in modern medicine over the past several years, pancreatic cancer is still associated with an extremely high mortality rate [1]. Currently, gemcitabine [3] and FOLFIRINOX [4] are considered first-line drugs for treating pancreatic cancer, but their efficacy is still low because of a seriously adverse reaction and because acquired drug resistance limits their cytotoxic efficacy. Therefore, we must find a novel target for enhancing current chemotherapy to improve the outcomes of patients with pancreatic cancer.

Resveratrol is a natural polyphenolic phytoalexin that is widely found in plants and in traditional Chinese medicines [16,17]. Resveratrol has been shown to directly inhibit the proliferation and viability of human pancreatic cancer cells in vitro in a dose- and time-dependent manner [22]. Accordingly, our results demonstrated that resveratrol inhibited cell proliferation and clone formation and induced cell apoptosis. Interestingly, we found that Panc-1 and BxPC-3 cells respond differently to gemcitabine (BxPC-3 being more sensitive). But their biochemical and functional response to resveratrol appears to be the same. As we know, BxPC-3 and Panc-1 cells have many differences such as K-*ras* mutation types (BxPC-3 is a K-*ras* wild cell while Panc-1 is a K-*ras* mutant cell) [34] and epithelial-mesenchymal transition (EMT) regulated genes (E-cadherin and Zeb-1) [35] which could affect gemcitabine sensitivity in pancreatic cancer cells. Our previous reports have shown that resveratrol could inhibit the EMT of pancreatic cancer cells via suppression of the PI3K/Akt/NF-kappaB pathway in Panc-1 and BxPC-3 cells [22]. Furthermore, resveratrol could also inhibit pancreatic cancer stem cell characteristics in human and Kras (G12D) transgenic mice by inhibiting pluripotency maintaining factors and epithelial-mesenchymal transition [36]. However, the specific mechanism needs to be further explored.

The AMPK system is traditionally considered a sensor of cellular energy status and a regulator of metabolism [37]. Recent studies have provided novel evidence that AMPK may function as a suppressor of cell proliferation. Indeed, activation of AMPK has been shown to benefit a variety of malignant tumors by inhibiting the proliferation of tumor cells [38,39]. In this respect, our results demonstrated that resveratrol could activate AMPK (Thr172) and suppress the proliferation of pancreatic cancer cells, indicating the anti-proliferative action of AMPK in pancreatic cancer cells. The effect of AMPK on cell proliferation appears to be mediated through multiple mechanisms, mainly the regulation of cell cycle progression, inhibition of protein synthesis, and de novo fatty acid biosynthesis [40,41]. Previous reports have suggested that AMPK activation stabilizes and increases AMOTL1 steady-state protein levels, contributing to YAP inhibition [42]. Accordingly, our results demonstrated that resveratrol induces YAP cytoplasmic retention and S127 phosphorylation and inhibits YAP transcriptional activity and YAP-dependent transformation via activation of AMPK. Knockdown of AMPK rescues resveratrol-induced suppression of YAP in pancreatic cancer cells. Some reports have suggested that glucose starvation and energy stress could result in phosphorylation of YAP and contribute to its inactivation [43]. Harris et al. [44] demonstrated that resveratrol could inhibit glycogen synthesis, which may serve as the underlying mechanism in the inhibition of YAP. However, Jung-Soon Mo [32] and his colleagues also found that AMPK inhibits YAP activity through phosphorylation of serine 94. Whether resveratrol could induce YAP phosphorylation at serine 94 need further investigation.

As an oncogene, YAP is abundantly expressed in many types of cancers [15,27], and tremendous progress has been made toward our understanding of the roles of YAP in tumorigenesis, size control, and stem cell renewal and differentiation [45,46]. However, YAP’s function in chemotherapeutic drug response is largely unknown in pancreatic cancer cells. Ciamporcero et al. [47] demonstrated that YAP overexpression protected, whereas YAP knockdown sensitized, urothelial cell carcinoma (UCC) cells to chemotherapy and radiation effects by increasing the accumulation of DNA damage and apoptosis. Verteporfin, a pharmacological YAP inhibitor, could inhibit tumor cell proliferation and restore sensitivity to cisplatin. In our study, we found that the chemotherapeutic sensitivity of pancreatic cancer to gemcitabine can be increased by YAP inhibition in vitro. YAP may participate in regulating the chemosensitivity of pancreatic cancer to chemotherapy. As a dietary and synthetic agent that is a pharmacological YAP inhibitor, resveratrol may exhibit greater efficacy and lower toxicity for the prevention and treatment of pancreatic cancer. However, its more specific mechanisms and safety require further investigation.

## 5. Conclusions

In conclusion, the present study demonstrated that resveratrol suppressed the proliferation and cloning ability and induced the apoptosis of pancreatic cancer cells. These multiple biological effects might result from the activation of AMPK (Thr172), thus, inducing YAP cytoplasmic retention and S127 phosphorylation, and inhibiting YAP transcriptional activity by resveratrol. YAP silencing by siRNA or resveratrol could enhance the sensitivity of gemcitabine in pancreatic cancer cells. These results suggest that resveratrol is a potential anticancer agent for the treatment of pancreatic cancer. However, whether other mechanisms are involved in the anti-tumor effects of resveratrol and its safety in humans warrant further study.

## Supplementary Materials

The following are available online at http://www.mdpi.com/2072-6643/8/10/546/s1, Table S1: A list of the utilized primary antibodies, Table S2: The siRNA sequences, Table S3: Primers sequences for real-time PCR analysis.

## References

[1] Siegel R.L., Miller K.D., Jemal A. (2016). Cancer statistics, 2016. CA Cancer J. Clin..

[2] Bosetti C., Bertuccio P., Negri E., La Vecchia C., Zeegers M.P., Boffetta P. (2012). Pancreatic cancer: Overview of descriptive epidemiology. Mol. Carcinog..

[3] Tamburrino A., Piro G., Carbone C., Tortora G., Melisi D. (2013). Mechanisms of resistance to chemotherapeutic and anti-angiogenic drugs as novel targets for pancreatic cancer therapy. Front. Pharmacol..

[4] Conroy T., Desseigne F., Ychou M., Bouche O., Guimbaud R., Becouarn Y., Adenis A., Raoul J.L., Gourgou-Bourgade S., de la Fouchardiere C. (2011). FOLFIRINOX versus gemcitabine for metastatic pancreatic cancer. N. Engl. J. Med..

[5] Edgar B.A. (2006). From cell structure to transcription: Hippo forges a new path. Cell.

[6] Huang J., Wu S., Barrera J., Matthews K., Pan D. (2005). The Hippo signaling pathway coordinately regulates cell proliferation and apoptosis by inactivating Yorkie, the Drosophila Homolog of YAP. Cell.

[7] Yimlamai D., Christodoulou C., Galli G.G., Yanger K., Pepe-Mooney B., Gurung B., Shrestha K., Cahan P., Stanger B.Z., Camargo F.D. (2014). Hippo pathway activity influences liver cell fate. Cell.

[8] Yu F.X., Zhao B., Guan K.L. (2015). Hippo Pathway in Organ Size Control, Tissue Homeostasis, and Cancer. Cell.

[9] Von E.B., Jaenicke L.A., Kortlever R.M., Royla N., Wiese K.E., Letschert S., McDuffus L.A., Sauer M., Rosenwald A., Evan G.I. (2015). A MYC-Driven Change in Mitochondrial Dynamics Limits YAP/TAZ Function in Mammary Epithelial Cells and Breast Cancer. Cancer Cell.

[10] Dubois F., Keller M., Calvayrac O., Soncin F., Hoa L., Hergovich A., Parrini M.C., Mazieres J., Vaisse-Lesteven M., Camonis J. (2016). RASSF1A Suppresses the Invasion and Metastatic Potential of Human Non-Small Cell Lung Cancer Cells by Inhibiting YAP Activation through the GEF-H1/RhoB Pathway. Cancer Res..

[11] Yagi H., Asanoma K., Ohgami T., Ichinoe A., Sonoda K., Kato K. (2016). GEP oncogene promotes cell proliferation through YAP activation in ovarian cancer. Oncogene.

[12] Wang J., Ma L., Weng W., Qiao Y., Zhang Y., He J., Wang H., Xiao W., Li L., Chu Q. (2013). Mutual interaction between YAP and CREB promotes tumorigenesis in liver cancer. Hepatology.

[13] Yuan Y., Li D., Li H., Wang L., Tian G., Dong Y. (2016). YAP overexpression promotes the epithelial-mesenchymal transition and chemoresistance in pancreatic cancer cells. Mol. Med. Rep..

[14] Morvaridi S., Dhall D., Greene M.I., Pandol S.J., Wang Q. (2015). Role of YAP and TAZ in pancreatic ductal adenocarcinoma and in stellate cells associated with cancer and chronic pancreatitis. Sci. Rep..

[15] Zhao Y., Khanal P., Savage P., She Y.M., Cyr T.D., Yang X. (2014). YAP-induced resistance of cancer cells to antitubulin drugs is modulated by a Hippo-independent pathway. Cancer Res..

[16] Borriello A., Bencivenga D., Caldarelli I., Tramontano A., Borgia A., Pirozzi A.V., Oliva A., Della R.F. (2013). Resveratrol and cancer treatment: Is hormesis a yet unsolved matter. Curr. Pharm. Des..

[17] Xu Q., Zong L., Chen X., Jiang Z., Nan L., Li J., Duan W., Lei J., Zhang L., Ma J. (2015). Resveratrol in the treatment of pancreatic cancer. Ann. N. Y. Acad. Sci..

[18] Zhang H., Morgan B., Potter B.J., Ma L., Dellsperger K.C., Ungvari Z., Zhang C. (2010). Resveratrol improves left ventricular diastolic relaxation in type 2 diabetes by inhibiting oxidative/nitrative stress: in vivo demonstration with magnetic resonance imaging. Am. J. Physiol. Heart Circ. Physiol..

[19] Hu F.W., Tsai L.L., Yu C.H., Chen P.N., Chou M.Y., Yu C.C. (2012). Impairment of tumor-initiating stem-like property and reversal of epithelial-mesenchymal transdifferentiation in head and neck cancer by resveratrol treatment. Mol. Nutr. Food Res..

[20] Buhrmann C., Shayan P., Popper B., Goel A., Shakibaei M. (2016). Sirt1 Is Required for Resveratrol-Mediated Chemopreventive Effects in Colorectal Cancer Cells. Nutrients.

[21] Roy S.K., Chen Q., Fu J., Shankar S., Srivastava R.K. (2011). Resveratrol inhibits growth of orthotopic pancreatic tumors through activation of FOXO transcription factors. PLoS ONE.

[22] Li W., Ma J., Ma Q., Li B., Han L., Liu J., Xu Q., Duan W., Yu S., Wang F. (2013). Resveratrol inhibits the epithelial-mesenchymal transition of pancreatic cancer cells via suppression of the PI-3K/Akt/NF-kappaB pathway. Curr. Med. Chem..

[23] Li W., Cao L., Chen X., Lei J., Ma Q. (2016). Resveratrol inhibits hypoxia-driven ROS-induced invasive and migratory ability of pancreatic cancer cells via suppression of the Hedgehog signaling pathway. Oncol. Rep..

[24] Qin Y., Ma Z., Dang X., Li W., Ma Q. (2014). Effect of resveratrol on proliferation and apoptosis of human pancreatic cancer MIA PaCa-2 cells may involve inhibition of the Hedgehog signaling pathway. Mol. Med. Rep..

[25] Ma Q., Zhang M., Wang Z., Ma Z., Sha H. (2011). The beneficial effect of resveratrol on severe acute pancreatitis. Ann. N. Y. Acad. Sci..

[26] Schmittgen T.D., Livak K.J. (2008). Analyzing real-time PCR data by the comparative C(T) method. Nat. Protoc..

[27] Hall C.A., Wang R., Miao J., Oliva E., Shen X., Wheeler T., Hilsenbeck S.G., Orsulic S., Goode S. (2010). Hippo pathway effector Yap is an ovarian cancer oncogene. Cancer Res..

[28] Basu S., Totty N.F., Irwin M.S., Sudol M., Downward J. (2003). Akt phosphorylates the Yes-associated protein, YAP, to induce interaction with 14–3-3 and attenuation of p73-mediated apoptosis. Mol. Cell.

[29] Dong J., Feldmann G., Huang J., Wu S., Zhang N., Comerford S.A., Gayyed M.F., Anders R.A., Maitra A., Pan D. (2007). Elucidation of a universal size-control mechanism in Drosophila and mammals. Cell.

[30] Lu L., Li Y., Kim S.M., Bossuyt W., Liu P., Qiu Q., Wang Y., Halder G., Finegold M.J., Lee J.S. (2010). Hippo signaling is a potent in vivo growth and tumor suppressor pathway in the mammalian liver. Proc. Natl. Acad. Sci. USA.

[31] Yi C.O., Jeon B.T., Shin H.J., Jeong E.A., Chang K.C., Lee J.E., Lee D.H., Kim H.J., Kang S.S., Cho G.J. (2011). Resveratrol activates AMPK and suppresses LPS-induced NF-kappaB-dependent COX-2 activation in RAW 264.7 macrophage cells. Anat. Cell Biol..

[32] Mo J.S., Meng Z., Kim Y.C., Park H.W., Hansen C.G., Kim S., Lim D.S., Guan K.L. (2015). Cellular energy stress induces AMPK-mediated regulation of YAP and the Hippo pathway. Nat. Cell Biol..

[33] Cao J., Yang J., Ramachandran V., Arumugam T., Deng D., Li Z., Xu L., Logsdon C.D. (2015). TM4SF1 Promotes Gemcitabine Resistance of Pancreatic Cancer in vitro and in vivo. PLoS ONE.

[34] Shao T., Zheng Y., Zhao B., Li T., Cheng K., Cai W. (2014). Recombinant expression of different mutant K-ras gene in pancreatic cancer Bxpc-3 cells and its effects on chemotherapy sensitivity. Sci. China Life Sci..

[35] Arumugam T., Ramachandran V., Fournier K.F., Wang H., Marquis L., Abbruzzese J.L., Gallick G.E., Logsdon C.D., McConkey D.J., Choi W. (2009). Epithelial to mesenchymal transition contributes to drug resistance in pancreatic cancer. Cancer Res..

[36] Shankar S., Nall D., Tang S.N., Meeker D., Passarini J., Sharma J., Srivastava R.K. (2011). Resveratrol inhibits pancreatic cancer stem cell characteristics in human and KrasG12D transgenic mice by inhibiting pluripotency maintaining factors and epithelial-mesenchymal transition. PLoS ONE.

[37] Foretz M., Taleux N., Guigas B., Horman S., Beauloye C., Andreelli F., Bertrand L., Viollet B. (2006). Regulation of energy metabolism by AMPK: A novel therapeutic approach for the treatment of metabolic and cardiovascular diseases. Med. Sci..

[38] Ming M., Sinnett-Smith J., Wang J., Soares H.P., Young S.H., Eibl G., Rozengurt E. (2014). Dose-Dependent AMPK-Dependent and Independent Mechanisms of Berberine and Metformin Inhibition of mTORC1, ERK, DNA Synthesis and Proliferation in Pancreatic Cancer Cells. PLoS ONE.

[39] Hadad S.M., Hardie D.G., Appleyard V., Thompson A.M. (2014). Effects of metformin on breast cancer cell proliferation, the AMPK pathway and the cell cycle. Clin. Transl. Oncol..

[40] Ma J., Duan W., Han S., Lei J., Xu Q., Chen X., Jiang Z., Nan L., Li J., Chen K. (2015). Ginkgolic acid suppresses the development of pancreatic cancer by inhibiting pathways driving lipogenesis. Oncotarget.

[41] Lin V.C., Tsai Y.C., Lin J.N., Fan L.L., Pan M.H., Ho C.T., Wu J.Y., Way T.D. (2012). Activation of AMPK by pterostilbene suppresses lipogenesis and cell-cycle progression in p53 positive and negative human prostate cancer cells. J. Agric. Food Chem..

[42] De Ran M., Yang J., Shen C.H., Peters E.C., Fitamant J., Chan P., Hsieh M., Zhu S., Asara J.M., Zheng B. (2014). Energy stress regulates hippo-YAP signaling involving AMPK-mediated regulation of angiomotin-like 1 protein. Cell Rep..

[43] Wang W., Xiao Z.D., Li X., Aziz K.E., Gan B., Johnson R.L., Chen J. (2015). AMPK modulates Hippo pathway activity to regulate energy homeostasis. Nat. Cell Biol..

[44] Harris D.M., Li L., Chen M., Lagunero F.T., Go V.L., Boros L.G. (2012). Diverse mechanisms of growth inhibition by luteolin, resveratrol, and quercetin in MIA PaCa-2 cells: A comparative glucose tracer study with the fatty acid synthase inhibitor C75. Metabolomics.

[45] Hong W., Guan K.L. (2012). The YAP and TAZ transcription co-activators: Key downstream effectors of the mammalian Hippo pathway. Semin. Cell Dev. Biol..

[46] Yu F.X., Guan K.L. (2013). The Hippo pathway: Regulators and regulations. Genes Dev..

[47] Ciamporcero E., Shen H., Ramakrishnan S., Yu K.S., Chintala S., Shen L., Adelaiye R., Miles K.M., Ullio C., Pizzimenti S. (2016). YAP activation protects urothelial cell carcinoma from treatment-induced DNA damage. Oncogene.

