# Supplementary material for: YAP Inhibition by Resveratrol via Activation of AMPK Enhances the Sensitivity of Pancreatic Cancer Cells to Gemcitabine
Source: Nutrients. 2016 Sep 23;8(10):546. doi: 10.3390/nu8100546 (PMC5083973; doi:10.3390/nu8100546)
Supplement: Supplementary file 1 [file nutrients-08-00546-s001.docx]

Supplementary Materials: YAP Inhibition by Resveratrol via Activation of AMPK Enhances the Sensitivity of Pancreatic Cancer Cells to Gemcitabine

Zhengdong Jiang, Xin Chen, Ke Chen, Liankang Sun, Luping Gao, Cancan Zhou, Meng Lei, Wanxing Duan, Zheng Wang, Qingyong Ma and Jiguang Ma

**Table 1.** A list of the utilized primary antibodies.

| **Antibody** | **Dilution & Use** | **Company** |
| --- | --- | --- |
| Rabbit anti-YAP | 1:2000 (WB)  1:150 (IF) | Abcam |
| Rabbit anti-AMPKα | 1:1000 (WB) | Cell Signaling Technology |
| Rabbit anti-p-AMPKα (Thr 172) | 1:1000 (WB) | Cell Signaling Technology |
| Rabbit anti-p-YAP (Ser 127) | 1:1000 (WB) | Cell Signaling Technology |
| Mouse anti-β-actin | 1:10,000 (WB) | Sigma |
| Goat anti-rabbit IgG-HRP | 1:10,000 (WB) | Abbkine. Inc |
| Goat anti-rabbit dylight 594  (red) IgG antibody | 1:150 (IF) | Abbkine. Inc |

**Table 2.** The siRNA sequences.

| **Genes** | **Primer Sequences** |
| --- | --- |
| siControl | sense: 5′-UUCUCCGAACGUGUCACGUTT-3′ |
|  | antisense: 5′-ACGUGACACGUUCGGAGAATT-3′ |
| siYAP | sense: 5′-GCUCAGCAUCUUCGACAGUTT-3′ |
|  | antisense: 5′-ACUGUCGAAGAUGCUGAGCTT-3′ |
| siAMPK | sense: 5′-UUCUCCGAACGUGUCACGUTT-3′ |
|  | antisense: 5′-ACGUGACACGUUCGGAGAATT-3′ |

**Table 3.** Primers sequences for real-time PCR analysis.

| **Genes** | **Primer Sequences** |
| --- | --- |
| YAP | Forward: 5′-TCCCAGATGAACGTCACAGC-3′ |
|  | Reverse: 5′-TCATGGCAAAACGAGGGTCA-3′ |
| Cyr61 | Forward: 5′-CAGGACTGTGAAGATGCGGT-3′ |
|  | Reverse: 5′-GCCTGTAGAAGGGAAACGCT-3′ |
| CTGF | Forward: 5′-CTTTGGCCCAGACCCAACTA-3′ |
|  | Reverse: 5′-GGCTCTGCTTCTCTAGCCTG-3′ |
| β-actin | Forward: 5′-AGCGAGTATCCCCCAAAGTT-3′ |
|  | Reverse: 5′-GGGCACGAAGGCTCATCATT-3′ |
